# Instructional Changes Adopted for an Engineering Course: Cluster Analysis on Academic Failure

**DOI:** 10.3389/fpsyg.2016.01774

**Published:** 2016-11-15

**Authors:** José A. Álvarez-Bermejo, Luis J. Belmonte-Ureña, África Martos-Martínez, Ana B. Barragán-Martín, María M. Simón-Márquez

**Affiliations:** ^1^Department of Informatics, Universidad de AlmeríaAlmería, Spain; ^2^Department of Economics and Business, Universidad de AlmeríaAlmería, Spain; ^3^Department of Psychology, Universidad de AlmeríaAlmería, Spain

**Keywords:** academic failure, metaphor, abstract concept, computer organization, concept metaphor

## Abstract

As first year students come from diverse backgrounds, basic skills should be accessible to everyone as soon as possible. Transferring such skills to these students is challenging, especially in highly technical courses. Ensuring that essential knowledge is acquired quickly promotes the student’s self-esteem and may positively influence failure rates. Metaphors can help do this. Metaphors are used to understand the unknown. This paper shows how we made a turn in student learning at the University of Almeria. Our hypothesis assumed that metaphors accelerate the acquisition of basic knowledge so that other skills built on that foundation are easily learned. With these goals in mind, we changed the way we teach by using metaphors and abstract concepts in a computer organization course, a technical course in the first year of an information technology engineering degree. Cluster analysis of the data on collective student performance after this methodological change clearly identified two distinct groups. These two groups perfectly matched the “before and after” scenarios of the use of metaphors. The study was conducted during 11 academic years (2002/2003 to 2012/2013). The 475 observations made during this period illustrate the usefulness of this change in teaching and learning, shifting from a propositional teaching/learning model to a more dynamic model based on metaphors and abstractions. Data covering the whole period showed favorable evolution of student achievement and reduced failure rates, not only in this course, but also in many of the following more advanced courses. The paper is structured in five sections. The first gives an introduction, the second describes the methodology. The third section describes the sample and the study carried out. The fourth section presents the results and, finally, the fifth section discusses the main conclusions.

## Introduction

In Hager (2008), the author discusses whether or not the learning process takes place only *inside* the student. When one refers to old theories of learning, two metaphors come to mind: the acquisition and the transfer metaphors.

To present the framework in which the paper was developed, we must first acknowledge that we, as human beings, are not able to think without using metaphors based on our life experiences or in what we have been taught. For example, when a toddler starts to explore and gain experience it learns via testing and then predicting the environment. After that, its first questions are asked of its relatives and those who must promote growth using simplistic metaphors. In other words, an adult may introduce a tiger as a wild cat, as the youngster probably only identifies with a household pet. Using this simplistic definition, the toddler can safely acquire new concepts, although this new knowledge will be refined later on. Using simplified contexts (Kövecses, 2015) always helps when facing more complex scenarios with no prior skills. In this first stage, metaphors are needed (Lakoff and Johnson, 1999; Hoover, 2016) to the case where the conceptual metaphors exposed by Lakoff and Johnson (1980) were not available. It is also considered that the process of making use of metaphors is involved in a creative process. Such a creative process is exploited, also, through metaphors to the tourism studies, as underlined in Adu-Ampong (2016) and discussed in Pereira de Barros et al. (2015).

Metaphors are useful but should not be used as a rule of thumb. In Scheﬄer (1960), a discussion on the fact that metaphors can also be counterproductive is presented. This led us to think that metaphors were used in the first stage of learning a complex concept or when knowledge must be acquired quickly and safely, meaning that the processing of the received information can be later used to synthesize the substratum upon which the coming knowledge will be built.

When we talk about learning, the mainstream thought is based, as Hager (2008) points out, on the fact that we learn because we first get the knowledge that we are going to need to solve problems and understand complex concepts. How to apply the knowledge is interesting in the field of engineering, but the most important part is the ability to acquire it and to extend it to connect with the knowledge that the student already had. Therefore, learning is built on how knowledge is acquired and the ability to apply it (Hager, 2005). The relevant part of cognition is embodied into the mind, and then we use our bodies to act accordingly. Following the line that Hager used to understand how we learn, Bereiter (2002) was the starting point considering that we may understand the mind as a recipient into which we could put content in during the process of acquisition.

Hager (2008) uses metaphors to explain the storyline, from Bereiter (2002) to Hager (2005), and these metaphors are the *acquisition metaphor* and the *transfer metaphor*. In Spain, the European Higher Education Space has considered moving from the traditional or formal educational model to a more dynamic one. The formal education model matches with the conception of the mind as a recipient and also defines the path to verifying if the student successfully acquired the knowledge via conventional tests or quizzes. We, at Universidad de Almería, understood this shift, imposed by EHES, in the educational paradigm as an opportunity to seize the metaphors used to understand learning. As Scheﬄer (1960) underlined, every metaphor has a limitation. Therefore, the way of teaching should also be shifted, accordingly. How sure are you that an exam, answered with no errors, means that all knowledge was properly acquired? From this first question, several others are also posed: Are we using models that tie content to a certain model of examination? Are we part of the academic failure of the students as we did not match the learning expectations? Is our teaching model providing concepts that easily adapt to a changing and rapidly evolving context?

Traditionally, the propositional learning, or static learning, was associated with higher education, whereas more practical learning and elemental versions of propositional learning were thought to be for low-profile students. In this model, if we analyze it, we are considering that the simplification of the context and the usage of the sensor-motor parts are catalysts to the acquisition of knowledge. But, is it correct that we prevent advanced students from using this schema of learning? We took this part to our analysis. We simplified the context and used more interaction to let freshmen acquire the key knowledge faster. Therefore, we tried to remove the first barrier of academic failure in engineering courses. We intended to homogenize skills at the first year. As in this learning schema, based in acquiring knowledge and applying it, there is an additional issue, which is the skill of acquiring the mentioned knowledge. This way we could consider that the mind is not the unique actor in learning as skills are not embodied into the mind. Skills are the tools that EHES-based educational models are using as their unit of learning (Hager and Holland, 2006). This is interesting from the perspective that skills are the result of learning as we have defined it. Let us consider that a certain student acquires some knowledge, and that he is able to transfer it by means of applying it to solve a set of problems. The student succeeded in that skill or competence. The EHES model is based in competences and, therefore, is not focused on the “things” the student knows, but rather on how they apply what they know. This is what is not considered in the conventional definition of learning, and it is a factor that affects learning (Hager and Smith, 2004).

If the conventional model fails in considering context (which is truly relevant, as exposed by Kövecses, 2015), then conventional teaching is not matching the needs and maybe it is not avoiding failure. Then what is learning? If we wish to reduce failure at universities, we should understand what learning is, and work to make the process of learning appropriate for this schema. Learning is defined by each one of us by tagging or constructing certain activities, like “learning” (Saljo, 2003). It is also affected by social-cultural issues, among others. This fact, of considering context as a key part of learning, is relevant using metaphors also (Kövecses, 2015).

How best to think of the process of learning and the ideal teaching techniques? (Hager, 2008) creates an interesting metaphor in which we have sustained an important part of our research. It was, in fact, in 2008 when we officially shifted our course methodology to the metaphor-based methodology, although the first course in which we started to apply metaphors was in 2005. The *becoming metaphor* considers that the learner is not disconnected from the context, and that has embodied knowledge, understanding, skills, and social context. This metaphor considers that learning is a process where the person and the context both change and adapt to one another (Beckett and Hager, 2002; Hager and Halliday, 2006).

If learning is understood using this definition, then any person can learn by means of their context, skills and understanding (Comellas-Carbó, 2015; Kövecses, 2015). When the students are in their first year of an engineering degree, the context is not suitable for the learning process as the student faces a new educative model and new concepts never seen before that must be quickly metabolized to acquire new concepts. If we accept that skills are almost homogeneously distributed over the student population that enrolls in Computer Organization, then we must make the process of acquiring new quick. Metaphors are a very interesting tool for this purpose as we simplify the context. Once we have done this, the knowledge interconnecting network can be enhanced soon with the newly acquired concepts. Reducing the time to metabolize the knowledge means being able to show new skills fast. These skills are needed to set the basis for following courses where it is assumed that skills are well assimilated.

The relevance of the metaphors in university teaching is obviously not limited to engineering and experimental sciences, but also applicable to all other areas of knowledge and job pursuit (Fuentes-González and Belmonte-Ureña, 2015).

The main objective of this paper is to present the advantages which come from using metaphors and abstractions as a teaching methodology for the Computer Engineering degree at the University of Almería. Thus, after the application of the technique based in the cluster analysis, a representative sample of the evaluations obtained by the students during the entire period comprising 11 different academic courses spread along 11 years, and focused on the course: Computer Organization (475 observations), it is clearly observed that two distinct groups, named Cluster 1 and Cluster 2, were found to be perfectly characterized.

## Methodology

Once we have set the framework where we intend to develop our methodology, we have to face an interesting and challenging task, which is teaching freshmen to get the needed skills. To do this, metaphors can help in the process of acquisition and contextualisation (Kövecses, 2015). Since the first stages of civilisation, the education process was based in the use of metaphors. They were useful to gain proximity with unrelated concepts. For example, in Margaret (2001) there is a clear example of how to use them to instruct library students. In that paper, authors use the trend outlined in Nibley (1991). There is a myriad of literature with respect to metaphors as a vehicle to knowledge (Sanders and Sanders, 1984), and as a means to reveal a path to the unknown. But, conveniently seized, metaphors can be quite useful (Rabinowitz, 1997).

There is an unavoidable cite when referring to metaphors; this is Lakoff and Johnson (1980), who presented the metaphor as an integral part of the way we see the world and of our “conceptual system.” According to Lakoff and Johnson (1980) and Hager (2008), metaphors are a key component of the human thought process. Each metaphor represents an underlying metaphorical concept that dictates the way we assume the context, or environment. The metaphors work because people in the culture understand the underlying concepts, even if they cannot articulate them (Comellas-Carbó, 2015).

It is widely accepted that learning can be explained by means of the *becoming metaphor* as defined in Hager (2008) and its relationship to embodied cognition, and that to ensure that learning is properly directed and achieved quickly, we can make use of metaphors. Wilson and Golonka (2013) discuss an interesting issue regarding metaphors and embodied cognition. Wilson and Golonka (2013) researched metaphors as a means to approach cognition. The interesting point revealed in their research has to do with the fact that metaphors are of interest for an embodied cognition field as they can map contextual and bodily experiences onto abstract concepts (Lakoff and Johnson, 1980). There is much literature consistent with the notion that conceptual metaphors inform and shape thinking (Landau et al., 2010). Conceptual metaphors are able to combine two concepts that intuitively fit together (Schneider et al., 2011). Using conceptual metaphors for thought and judgment can be better organized as they represent general mappings. The aspect, as revealed by Schneider et al. (2011), with conceptual metaphors that we have exploited is that they meet inferences that can reinforce an individual’s cognition. This is because one source concept activates another related concept.

Identifying target concepts and their relationships is the first step toward selecting the source concepts and the metaphors to establish the activation between source set and target set (Hellmann et al., 2013).

Using metaphors to activate target concepts from certain and previously selected source targets recalls the internal workings of a neural network, where connectivity is a key part of the embodied conceptual metaphor. When the usage of a conceptual metaphor is accepted and that it is part of the embodied cognition in the sense of the *becoming metaphor* (Hager, 2008), then experimenting results from the embodied cognition literature is easy. This can be done by adopting a computational model to create a controlled context where the source and target concepts are activated experimentally, in this way understanding how sensory motor mechanisms could emplace higher-level cognitive behavior over the process of learning. In Flusberg et al. (2010), authors create a controlled neural network to demonstrate the principle that gives birth to the *becoming metaphor*.

During the course of time, researchers have devoted their work to proving that the mind is situated and embodied. But how does cognition appear? It does appear through the physical interactions with the context, which is exactly what is stated by the *becoming metaphor*, the guiding principle that we are following to adopt the turn in our teaching. This is supported by researchers such as Clark (1998), Barsalou (1999, 2008), Lakoff and Johnson (1999), and Chemero (2009). Embodied cognition states that cognitive processes are tuned and structured by the interaction and reciprocal evolution of an agent and its context. In Rizzolatti and Craighero (2004), for example, emotion and action perception are evident in the cognition process (recently demonstrated by the work presented by Aziz-Zadeh and Gamez-Djokic, 2016). If we refer to Margaret (2001), the engagement through emotion was part of the success of the learning process, which is more proof that the *becoming metaphor* is useful to model learning. If it is useful to model learning, then the action of teaching can be directed to that model of learning and focusing in the pedagogy, as in Harr et al. (2014). If we aim to use metaphors to activate target concepts using source concepts, given that the mind and cognition are dependent and related to the context, then we can get to the point where we wonder if this mechanism is artificial. But, again, this is a debate that is continuously held between researchers (Lakoff and Johnson, 1980, 1999; Gibbs, 2006; Feldman, 2006; Pinker, 2007). It was Lakoff and Johnson (1980) who first proposed a widely accepted argument in favor of talking about complex or abstract concepts, the inevitable need for metaphors and borrowing elements from concrete and well-known domains. Therefore, metaphors are elements that we use not only when we talk about abstract things, but how we think of them as well.

The justification of the turn in our teaching methodology is experimentally demonstrated in the form of experiments showing that activating a concrete source domain influences inferences in the abstract target domain (Jostmann et al., 2009; Ackerman et al., 2010).

In Flusberg et al. (2010), the authors propose a controlled and simplistic model where these theories can be validated. This controlled model is a computational model based in neural networks. These simplified models let researchers deeply understand complex cognitive processes, to a point where embodied cognition was lacking (Spivey, 2007; McClelland, 2009).

## Context

The context where the methodology based in understanding the learning process described in Hager (2008), by the *becoming metaphor*, where the cognition can be approached by means of interconnected concept metaphors, was applied in a Computer Organization course, which is scheduled during the first semester of the first year. In this course, the knowledge of the students is void as it is mostly technical. The context is therefore not helping the learning process, if we understand it using the *becoming metaphor*. In addition to this, students—freshmen—have enrolled in a degree where a high percent of the curricula was not studied in previous stages. In this context, it is vital that the knowledge that the student acquires is properly achieved and metabolized as it is the foundational framework for all the concepts required in upcoming courses. Also, it guarantees that skills and competences are properly acquired.

During the Computer Organization course the student faces a very low level of details in terms of how the computer is designed. This requires the student to be engaged in the understanding of all details. Many of the concepts are directly used by other courses, even during the same semester. These include the concepts of cache memory and the concept of system clock. Others are used in more advanced courses, like the concept of micro-architecture and instruction-set architecture. So, if we fail to properly introduce this knowledge at this stage, then the failure (and sentiment of low engagement) will be a constant during the students’ career. Recall that, according to Rizzolatti and Craighero (2004), emotions are an important part of the learning process and of cognition. Here is where we adopted the methodological change to reduce failure, at least by means of metaphors to let students land smoothly in this course, feel confident with the contents, and therefore promote their skills.

**FIGURE 1:**
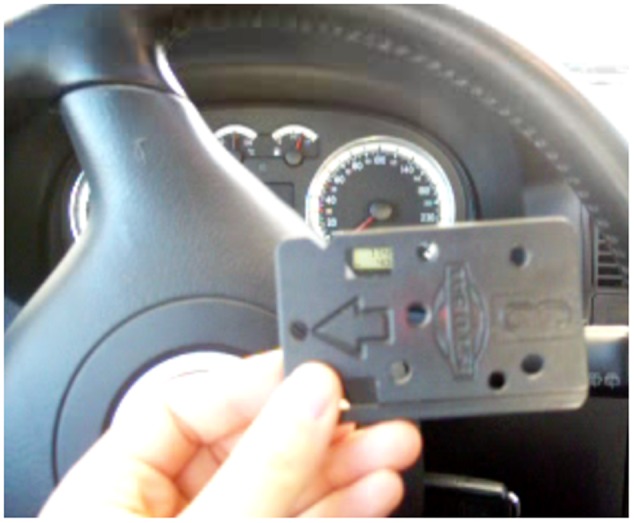
**Plastic token as a metaphor for the instruction**.

**FIGURE 2:**
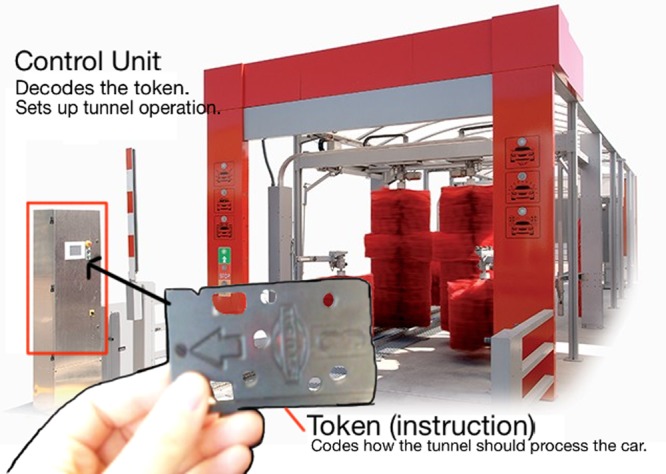
**Small device that reads the token and configures the tunnel.** Metaphor for control unit and instruction decoding.

**FIGURE 3:**
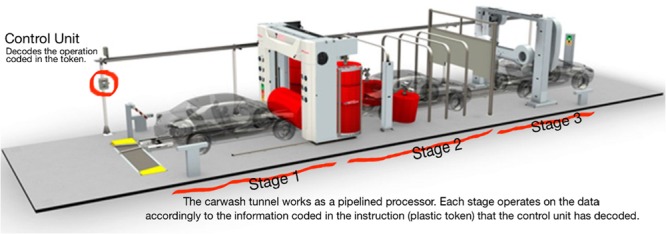
**Car-wash tunnel metaphor for the data-path unit of a processor**.

**FIGURE 4:**
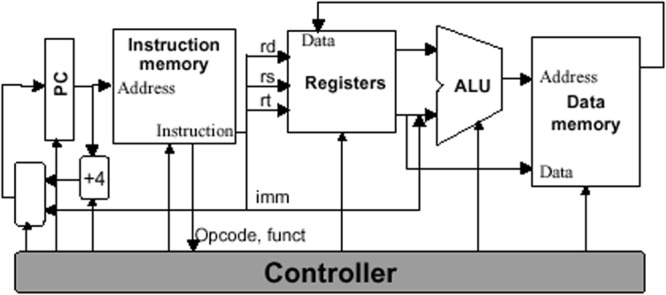
**Simplistic model of the processor architecture**.

**FIGURE 5:**
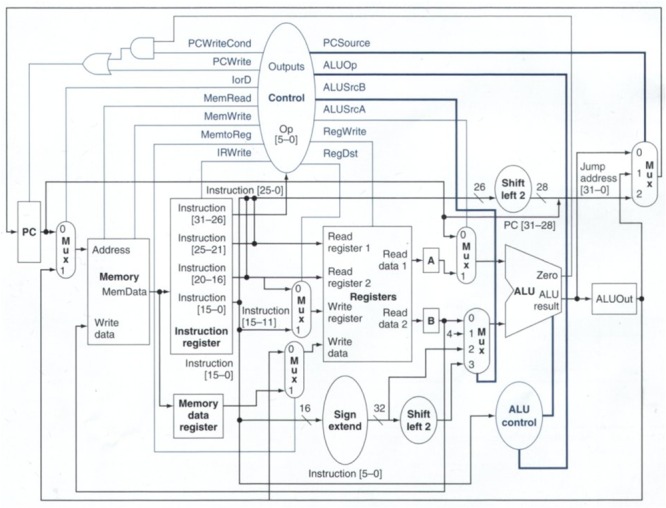
**Final model of the processor architecture**.

The course contents were completely redesigned to accommodate the changes referring to the mentioned concepts: cache, system clock, and micro-architecture and instruction-set architecture. We moved from the mere transmission of technical details to a more PPK (Pedagogical Psychological Knowledge) centered method where the metaphors were the core part:

(i) *Understanding cache*: The cache memory of the system, in Computer Organization, was previously defined as an internal memory built with fast semiconductors that can be found both inside and outside of the core in the chip-set IO processor. If a student in his first semester is given this, then the negative emotional impact may affect the rest of the learning process. But, if the concept is rewritten as: You may understand the cache as an internal memory that resides inside the processor core and in the auxiliary communications processor. Its purpose is making data processing quicker, so to understand the cache means thinking of your kitchen pantry or fridge. If you do not have cache, or a pantry, then you will not eat until you go to the grocery store, get home, cook, sit down, and then finally eat your first course. To eat your second dish, you need to stand up, return to the supermarket (your main memory area), and do everything all over again. What about dessert? Again, you leave your home (processor), go to the supermarket (main memory), get your ice cream (data), return home, and eat it (process it). How may this affect your cache (first dish, second dish, dessert)? If you use your cache (pantry) when you visit the supermarket you get all the data that you may need for your next few operations, and keep that in the pantry. So when you pretend to have lunch, your first dish is already there. You cook it so you can proceed directly to the second dish without having to leave your home (processor). This accelerates your process of eating. The pantry is small in size (you do not really need a big one) but many foods have expiration dates. Size and expiration dates are two concepts that are related to: temporal locality and spacial locality that affects program performance. If your pantry is big you may store data (food) that you will never eat and then dispose of due to your expiration date. So, you and the processor, when using the cache, are filled up with little data. These data are the information that the processor will cook in the next operations (days)—temporal locality—and these pieces of data are related and tied to a date, like food.
(ii) *Understanding the system clock*: It is in this course (Computer Organization) where the importance of the quartz oscillator is revealed. The oscillator is the system clock. It commands the speed at which every part of a processor can work. The problem here is that the number of students that really got the meaning and the importance of the oscillator was very small when the definition given was this: *The oscillator is a train of squared digital pulses*. Students tend to study its definition and repeat exercises until, mechanically, they were able to “solve them.” If the exercise was changed, the student did not know how to do it. In this case we used the metaphor of the galley. In it there is a person that sets the working rhythm by hitting a drum. Each time the person hit the drum, all the rowers had to row at the same time. Otherwise, the galley would not advance in a straight line. If the person that hits the drum increases the frequency of the hits, then rowers work faster, the galley travels faster. Also, related to the frequency concept, we can introduce concepts such as the heat generation and loss of performance (as the drummer increases his frequency, rowers will operate faster, and therefore be affected by the heat they generate; if the galley is not able to reduce the heat, then rowers will throttle down to avoid collapse). The drummer is the system clock and with this concept we can reveal related and abstract concepts as frequency and its relationship to performance, the heat problem and the importance of using the correct heat sink (TDP factor) to avoid collapse or a loss of performance due to a frequency deceleration. These concepts can be perfectly revealed and prepared if the metaphor is explained instead of immediately using the technical definition, which will be perfectly understood once the metaphor is transferred to the class.
(iii) *Entering into a real person-sized processor*: It is also in this course where the students are told about a program and the fact that it is composed by instructions. The instructions are pieces of 1s and 0s that tell the processor which circuit should be used (adders, multipliers, etc.) and with which data to do all the tasks that are implemented into a program. Concepts such as the ISA (Instruction Set Architecture) are also presented in this course. As you may see, there are many new and technical concepts that harness skill acquisition. For these concepts we prepared the metaphor of the car-wash tunnel, where the inner workings of a processor are revealed comparing the architecture of a processor to that of a car wash tunnel. In a first instance, the students are presented *the instruction* that is explained using the plastic token (**Figure 1**) that car-wash tunnels use to operate. This plastic token has little holes which let the car-wash tunnel read the type of washing operation you bought. This works exactly the same as with computer instructions where 1s and 0s configure the type of operation to be done with the data. In this case, the data is our car. In computer instruction it is, for example, a number. The plastic token is inserted in a small device (**Figure 2**) that reads the token and prepares the whole architecture of the tunnel for you. The same occurs in a processor. The instruction is decoded in the *control unit*. This device that understands the plastic tokens is key for the processor. This device sets the number of different instructions according to the different operations that it is able to do. This set of instructions (plastic tokens) is what we call the Instruction Set Architecture. Two processors that have the same *small device*, without caring about the rest of the architecture, are compatible (Intel and AMD). The rest of the car-wash tunnel is what (in processor) is called data path (**Figure 3**). This is where data (cars) pass and is operated according to the instruction (plastic token) that we have inserted into the car-wash tunnel (control unit). **Figure 4** shows the technical sketch to explain all these concepts. Students are now presented this metaphor and then they are given the technical sketch. **Figure 5** shows the target skill that the student must acquire. We see that if the students are offered the technical concepts with no metaphors, they take much more time to get the target skills. If they are exposed to the metaphors, they quickly understand the concepts and are able to advance faster.

As a direct consequence of the methodological change in this context (it was started in 2005), we experienced an improvement in results. We decided to switch textbooks from the conventional one to one more focused on the concept of metaphors. Since then, the recommended textbook for this course is Alvarez-Bermejo (2008). This book has redesigned the course without compromising the curriculum and has also helped first year students to adapt to the material more quickly.

## Method

To have a real contrast of the effectiveness of the instructional changes adopted, instead of simply evaluating the performance of each student, we have considered that to add their opinion as well as input from students themselves is of relevance. For this purpose, a table has been built for each student, including data that identify the course (year, group, etc.), date on which the student took the exam, final grades, and the percentage of homework/labs fulfilled by the student during the course and the attendance rate. Additionally, we also took as a reference the feeling of the students (their opinion); in our university we, the faculty, have access to the evaluation that the student completes when the course is over. This evaluation, controlled by our university, is completed by the student (as a means to measure teaching quality). The data related to these surveys, which are passed out to the students each semester, are provided to us. These surveys are collected while gathering student evaluations, for each teacher and course, during that semester and at the end of the period. However, as each survey completed by any student is anonymous it is not possible to obtain a certain student’s answer. Therefore, the data are aggregated to build a performance profile for each faculty. To incorporate the results of such surveys, we are using the average opinion for each course. Thus, the sample consists of 475 records, which reflect the performance and behavior that every student has had regarding the Computer Organization course for the last 11 academic years, i.e., from 2002/2003 to 2012/2013.

For each student six variables are defined:

V1: Identification number (ID) for each student. This is unique and personal.
V2: Academic course in which the record of the student was obtained.
V3: Examination date in which the student opted to take the examination; this date can take four possible values: February, June, September, or December.
V4: Qualification obtained for the course. This variable shows the numeric value representing the success value to pass the course.
V5: Percentage of lab assignments that the student was able to complete. This percentage is accounted as additional to the written exercise (exam) and therefore adds to the final score of the student. This additional evaluation is considered, somehow, a continuous evaluation to evaluate how the student progresses.
V6: Number of absences, or rate of absenteeism, in the lecture sessions, which includes how often the student missed lectures during the semester. This entry gives an idea of the degree of involvement, engagement, and student interest in the course.

It is also known that during the 11 academic courses analyzed and sampled, the teacher of the course was the same. The unique, significant turn in the methodology used ordinarily for the course was the adoption, from the academic year 2005/2006, of a new method of lecturing that was supplemented with notes of support in the form of metaphors and abstractions following, first, the embodied cognition metaphors and from 2008 the *becoming metaphor* to understand learning. The resulting course material was subsequently published as a book (Alvarez-Bermejo, 2008) that is still in use (and whose edition was extended to Latin America).

**Table 1** gathers a summary of the sample under study, examination dates, and average scores.

**Table 1:** Distribution of the simple: course, examination date and average scores.

Course	Exam date	Students	Average score	% Labs assigments completed	Average absenteeism
2002–2003	december	15	3,60	26%	2,10
	june	51	3,29	23%	2,31
	september	24	2,89	19%	2,50
2003–2004	december	18	5,61	46%	1,00
	june	46	4,25	33%	1,95
	september	12	5,04	40%	1,42
2004–2005	december	8	5,75	48%	0,88
	june	33	3,82	28%	2,36
	september	18	3,83	28%	2,33
2005–2006	december	6	4,67	37%	1,67
	june	20	5,94	53%	0,97
	september	10	4,20	67%	2,45
2006–2007	december	3	7,50	100%	0,25
	june	39	5,05	76%	1,94
	september	13	5,77	83%	1,40
2007–2008	december	3	5,00	75%	1,92
	june	35	5,98	85%	1,33
	september	10	6,17	87%	1,14
2008–2009	diciembre	3	6,00	85%	1,25
	junio	34	4,88	74%	2,04
	septiembre	9	6,00	85%	1,25
2009–2010	june	30	5,33	78%	1,70
	september	4	6,00	85%	1,25
2010–2011	june	12	3,00	55%	3,25
	september	8	6,00	85%	1,25
2011–2012	february	1	6,00	85%	1,25
	june	8	5,00	75%	1,92
	september	1	6,00	85%	1,25
2012–2013	february	1	6,00	85%	1,25
	june	1	7,50	100%	0,25

The mining of information gathered from the sample was started with an analysis of the characteristics of the group of students surveyed through the use of statistical classification techniques and cluster analysis, which allowed us obtain a characterisation of the students evaluated into two clearly separated groups.

The cluster analysis used for the processing of information consisted of the application of the K-means algorithm. However, in a previous stage, the DBSCAN—Density-Based Spatial Clustering of Applications with Noise—proposed by Ester et al. (1996) was applied. Both methods (K-means and DBSCAN) have been used in numerous research papers as a path to obtain the optimal number of groups that can be created from the original sample. So, the latter algorithmic procedure provides a significant advantage over the classical cluster analysis based on the K-means algorithm, because with DBSCAN it is not required to specify the number of final clusters desired.

## Results

The realization of the cluster analysis has allowed us to obtain deeper knowledge of the characteristics of the two groups of students that make up each cluster. We have considered three typifying variables from each cluster, as presented in **Table 2**.

**Table 2:** Typifying variables from each cluster and descriptive statistics.

Variable	Description	Mín.	Max.	Average	Typical deviation	Variation coefficient
V_4_	Final grade score for the student	2,00	10,00	4,51	1,99	44,0%
V_5_	Percentage of lab assignments completed	10%	100%	48%	0,30	62,3%
V_6_	Absenteeism rate for lecture sessions	0,00	3,25	1,89	1,12	59,3%

In view of the statistics, the analysis regarding the coefficient of variation (CV) is remarkable. This statistical concept is used to instrument the dispersion of data regardless of the units in which the variable is expressed. Thus, the higher the CV, the greater the dispersion, that is, less data homogeneity. In this regard, it is noteworthy that the high dispersion was presented by V5 (percentage of lab assignments completed by the student), with a CV value of 62.3% and the rate of absenteeism registered during the lecture sessions (V6).

Thus, the total sample consisting of 475 students that took the examination and were qualified for the period comprising the eleven academic courses have been classified into two homogeneous groups: one with 257 records (Cluster 1) and the other with 218 (Cluster 2). Only three from the total of six variables considered for the study were instrumental in breaking this classification into two clusters, as presented in **Table 3**.

**Table 3:** ANOVA analysis of the typifying variables.

Variable	Descripción	aggregated cuadratic mean	gl	Error cuadratic mean	gl	*F*	Sig. (^∗^)
V_4_	Final grade for the student	1593,422	1	0,585	473	2723,210	0,0000000
V_5_	Percentage of lab assignment completed by the student	25,307	1	0,038	473	657,883	0,0000000
V_6_	Rate of absenteeism in lecture sessions	545,007	1	0,110	473	4944,834	0,0000000

*(^∗^) At the 95% of confidence, all the typifying variables are significant.*

According to the average of the records registered for the six variables, the characteristics of each cluster are described as:

**Cluster 1**. “Unmotivated students with the course.” There were 257 students, since the academic year 2002/2003 until mid-2004/2005, who enrolled in the course Computer Organization when the teaching of the lectures was without any methodological change adopted other than the conventional teaching/learning scheme. This group is characterized in that it has an average of qualification (V4) below the overall average, that is, 3.88 compared to 4.51. In both cases, the score is a signal of academic failure. This group is also characterized as the least participative, with a very low level of engagement in class with a very low number of lab assignments completed, which is a factor that affects negatively on continuous evaluation delivered as only 28.75% of the total of labs assignments scheduled by the faculty. Finally, this group of students is the group with the highest average number of absenteeism during lecture hours, with two absences per student on average.
**Cluster 2**. “Motivated students with the course.” This cluster brings together 218 students who were learners by applying the methodology of using metaphors and abstractions to design new study material and additional study material to the conventional contents of the course. Specifically, they were given the concepts in the way described in section context previously defined. This group is characterized in that it has an average of qualifications (V4) higher than the overall average, that is, 5.26 compared to 4.51, reflecting a considerable increase in the evaluation of the group. This positive result in final grades is reinforced by the high participation of students in class as students grouped in this cluster finished the final exam with a high percentage of their lab assignments completed (70.91%). Finally, it is worth noting that the percentage of absenteeism during lecture hours has been successfully reduced, as the engagement of the students was much greater than Cluster 1.

**FIGURE 6:**
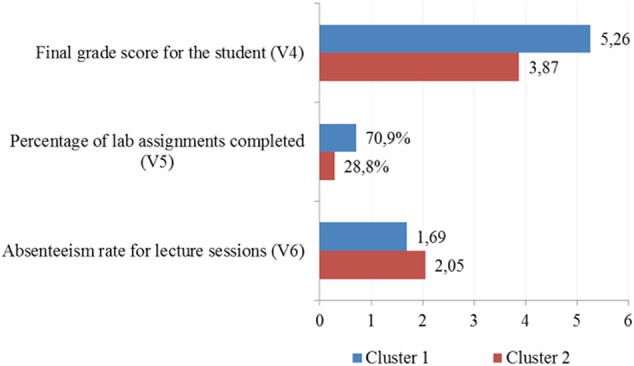
**Characterisation of the cluster variables**.

**Table 4** and **Figure 6** show the behavior of each variable, its mean value, according to the cluster considered.

**Table 4:** Cluster characterization.

	Num observations	Final grade V_4_	% completed labs V_5_	Num. absenteeism V_6_
Cluster 1	257	3,87	28,75%	2,05
Cluster 2	218	5,26	70,91%	1,69
Sample	475	4,51	48,16%	1,89

## Discussion

There is a myriad of methods developed in infinite literature on teaching students critical thinking skills and also on how to involve them in the learning process. The methods applied, combined with the advanced concepts, made for a more interesting instruction session for all involved. The success of the method in this subject has encouraged us to experiment with courses for sophomores. The result was students who were able to more quickly absorb the base concepts in which they needed to build superior knowledge. We achieved a twofold objective: On one hand, we could reduce the percentage of failure without losing quality and depth in the course. On the other hand, we were able to discover that students felt self-confident as they could successfully deal with the subject.

The results of this work show that the application of metaphors and abstractions as additional support to the conventional methodology followed during conventional lectures is useful to enhance the academic performance, engagement, and motivation of many students. This was proven by the increased participation in class and the reduction in the ratio of absences per student. The cluster analysis technique has allowed the objective detection of two groups of students: unmotivated and motivated, raised from performance that they have shown after analyzing their final grades, the percentage of completed lab assignments, and the number of absences from lecture hours.

When evaluating possible teaching strategies and methodologies, especially for the first courses in technical careers that intend to improve the student motivation and engagement and reduce academic failure at university, it is interesting to consider the advantages offered by metaphors to enhance student learning abilities and the acquisition of skills and competences that are planned in their respective curricula.

## Author Contributions

JÁ-B, LB-U, ÁM-M, AB-M, and MS-M designed the study. Dr. JÁ-B was in charge of collecting all the data from each course and student during the whole period. And describing the teaching strategies for each one of the years of the study. The description of each metaphor and concept that changed during the course instruction was also carefully documented. Dr. LB-U designed all the data analysis related to the collected data. His knowledge of the cluster analysis techniques (usually applied to the labor market) was of real help for getting numerical patterns and figures. The data, in fact, were treated following the same guidelines as in labor markets, in fact. ÁM-M interpreted the data together with Dr. LB-U, from the methodological perspective. Her support was relevant as data should be interpreted in the field of metaphor thinking and learning. AB-M and MS-M, both provided all the methodological support to the study to understand how the instructional changes affected in improving the success rate of the students. And who framed the changes into the concept of metaphors. All the authors were active during the edition of the paper.

## Conflict of Interest Statement

The authors declare that the research was conducted in the absence of any commercial or financial relationships that could be construed as a potential conflict of interest.

The reviewer RC-M and the handling Editor declared their shared affiliation, and the handling Editor states that the process nevertheless met the standards of a fair and objective review.
